# Seizure localization using pre ictal phase-amplitude coupling in intracranial electroencephalography

**DOI:** 10.1038/s41598-019-56548-y

**Published:** 2019-12-27

**Authors:** Nuria E. Cámpora, Camilo J. Mininni, Silvia Kochen, Sergio E. Lew

**Affiliations:** 1 Centro de Epilepsia, Hospital Ramos Mejía. Estudio en Neurociencias y Sistemas Complejos (ENyS),CONICET. Universidad de Buenos Aires, Buenos Aires, Argentina; 2 Instituto de Biología y Medicina Experimental – CONICET, Buenos Aires, Argentina; 3 Instituto de Ingeniería Biomédica, Universidad de Buenos Aires, Buenos Aires, Argentina

**Keywords:** Neuroscience, Epilepsy

## Abstract

Understanding changes in brain rhythms provides useful information to predict the onset of a seizure and to localize its onset zone in epileptic patients. Brain rhythms dynamics in general, and phase-amplitude coupling in particular, are known to be drastically altered during epileptic seizures. However, the neural processes that take place before a seizure are not well understood. We analysed the phase-amplitude coupling dynamics of stereoelectroencephalography recordings (30 seizures, 5 patients) before and after seizure onset. Electrodes near the seizure onset zone showed higher phase-amplitude coupling. Immediately before the beginning of the seizure, phase-amplitude coupling dropped to values similar to the observed in electrodes far from the seizure onset zone. Thus, our results bring accurate information to detect epileptic events during pre-ictal periods and to delimit the zone of seizure onset in patients undergoing epilepsy surgery.

## Introduction

Brain rhythm variability has been found not only in many brain state transitions, including wakefulness, drowsiness and sleep^1^, but also in epilepsy, especially during the onset of epileptic seizure^2^. In a first interaction level, changes in brain rhythms can be detected by looking at the power of different frequency bands. On a second interaction level, brain rhythms are dependent among themselves, with interactions and modulations between frequency bands^3^. It has been shown that the phase of lower frequencies is locked with changes in amplitude of higher ones, and that this phase amplitude coupling (PAC) can be measured by means of the Phase Locking Value (PLV)^4,5^.

The seizure onset zone (SoZ) is defined as the site of “primary organization” of ictal discharge. Therefore the “frequency spectrum as well as the interareal synchronization at seizure onset constitute a pattern of organization that is reproducible from one seizure to another”^6^, and its localization is critical for successful surgical treatment. In this regard, measuring PAC has been useful for SoZ localization^7^. For example, second order interactions between low frequency phase and high frequency amplitude has been more useful to identify the SoZ than the power of high frequency bands alone^8^. In the same line, PAC between beta and high gamma resulted in a better predictor of ictal states than the amplitude of high gamma alone^9^. In Weiss *et al*., (Neurology, 2015), authors reported that if at least 75% of early phase-locked high gamma sites were included in the resection area, the outcome improves, giving some insights on how to use their coupling measure to efficiently resect brain tissue during surgery. Motoi *et al*. found that the strength of PAC between high-frequency activity (>150 Hz) and the phase of slow waves (3–4 Hz) predicted surgery outcome^10^.

While phase locking between low frequencies (2–25 Hz) and high gamma (80–150 Hz) increased after seizure onset^2^, slow wave driven modulation of high gamma was identified during preictal periods in some epileptic patients^11^. In addition, decreasing synchronization values between near electrodes were found previous to the seizure onset^12^.

Studying the activity that precedes ictal onset is appealing because it may reveal the mechanisms that trigger the seizure. In this regard, the goal of the present work was to study the dynamics of PAC phenomena before and during the seizure, by computing PLVs among many frequency bands in stereoelectroencephalography (SEEG) recordings.

## Materials and Methods

This study was approved by the Review Ethics Board of El Cruce Hospital, Buenos Aires, Argentina, according to the Declaration of Helsinki. All patients were informed of the purpose and possible consequences of this study and signed an ethical board-approved written informed consent.

### Data

We analysed SEEG recordings from five patients who underwent presurgical evaluation at High Complexity Hospital El Cruce “Nestor Kirchner”, Florencio Varela, Argentina, between 2013 to 2015.

Demographic and clinical data are provided in Table 1. We included 5 patients and 30 seizures. We determinate the hemisphere (left/right) where seizures began based on semiology, inter ictal discharge, the beginning of the ictal discharge, magnetic resonance imaging, and the information of PET results (see Table 1).

**Table 1:** Demographic characteristics of the patients included M: male, F: female, MRI: Magnetic resonance image; PET: Positron emission tomography.

Patient	Sex/age (years)	Ictal Semiology	MRI	PET	Total number of contacts in depth electrodes	Numbers of seizures analysed	Localization of deep electrodes	Epileptogenic Zone	Hemisphere (Epileptogenic Zone)	Engel	Follow up
1	F/22	paraesthesia, bitter taste in the mouth, sharp sound and alteration of consciousness	normal	diffuse right frontal cortical hypometabolism	44	5	Right hippocampus, amygdala and insula	Insula	Right	II	5 years
2	M/37	alteration of consciousness, immobility, oral and right hand automatisms	normal	left temporal hypometabolism	54	6	Hippocampi bilaterally	Mesial temporal	Left	II	2 years 8 months
3	M/19	deja vu, alteration of consciousness, immobility and swallowing automatisms.	normal	mild hypometabolism bitemporal	54	4	Hippocampi bilaterally	Mesial temporal	Left/Right	No surgery yet	N/A
4	M/28	speech arrest, fixed gaze, alteration of consciousness, immobility, slow right oculocephalic deviation. Some seizures were with secondarily generalized	Frontal cortical dysplasia	not performed	45	6	Frontal lesion	Frontal medial	Left	I	3 years
5	M/33	cephalic and ocular version to the right, deviation of the right labial commissure and aphasia of expression. Some seizures were with secondarily generalized	Frontal cortical dysplasia	not performed	52	9	Frontal lesion	Frontal lateral	Left	II	2 years

An average of 49 contacts of depth electrodes per patient (range: 44–52) was implanted using an image-guided system. We analysed all the available electrodes with the exception of contacts with major artefacts.

### Electrodes Implantation

Depth electrodes (Ad Tech) had: (a) 8 or 10 platinum contacts with 5 or 10 mm inter-contact center to center distance, contact length of 2.4 mm and 1.1 mm diameter, or (b) 9 platinum contacts with 3 mm distance between the first and the second contact and 6 mm inter-electrode distance from the second to the last. Contact length was 1.57 mm and the electrode diameter was 1.28 mm. Electrodes were identified by a letter of the alphabet. There is no standard labelling for each location. Contacts within an electrode are usually identified with numbers beginning from the deepest (contact number 1, corresponding to the tip) to the base.

### Anatomic localization of electrode positions

Electrode placement considered not only the final target but also the precise trajectory. Surgical planning of electrode placement in specific anatomical structures was thus dependent on the pre-established hypotheses of probable zone of seizure onset and propagation. Preimplantation hypotheses including electroclinical and MRI data were determined by two epileptologists (NC and SK) based on seizure semiology and interictal and ictal findings of scalp video-EEG, MRI (3.0-T), PET (in Patients 1,2 and 3) together with neuropsychological and psychiatric evaluation^13^. Therefore, electrode positions were not standardized.

### Intracranial electroencephalography recordings

Intracranial electroencephalography (iEEG) signals were acquired using the software Cervello 1.04.200, sampled at 2000 Hz with bandpass filtering between 0.7 Hz and 200 hz. The seizure onset was identified by two epileptologists (NC and SK) through independent reviews. Ictal onset was defined as initial iEEG changes, characterized by sustained rhythmic discharges or repetitive spike-wave discharges that cannot be explained by state changes and that resulted in habitual seizure symptoms similar to those reported in previous studies. The seizure onset zone (SoZ) was defined as the contacts where the earlier ictal iEEG changes were seen. These contacts, which evidence epileptic discharges at any moment of the seizure, were defined as “involved”. Those which did not evidence epileptic activity were defined as “not involved”.

The resection area was determined based on the iEEG findings and other pre-surgical evaluations.

### Data analysis

Recordings were downsampled to 500 Hz. The phase locking value^4^ in a determined time window was computed as:

1 $$PLV=\frac{1}{N}\,|\mathop{\sum }\limits_{n=1}^{N}{e}^{-i({\phi }_{H}(n)-{\phi }_{L}(n))}|$$

where *N* stands for the total number of samples in the selected window of analysis, ${\phi }_{H}(n)$ is the phase angle at sample *n* of the envelope of the high frequency band, and ${\phi }_{L}$ is the phase angle at sample *n* of the low frequency band. Before computing PLV, we filtered the down-sampled signal using a zero-phase filter in order to obtain low and high frequency bands. To compute ${\phi }_{H}$ we first obtained the envelope of the high frequency band as the amplitude of its Hilbert transform. Then, we computed the Hilbert transform once again over the envelope and took its phase angle as ${\phi }_{H}$. To obtain ${\phi }_{L}$ we computed the Hilbert transform of the low frequency band and took its phase angle.

For our analysis we computed the PLV in 1 second windows. Low and high frequency bands were obtained by filtering the down sampled recording around the target frequency ± 0.5 Hz, as previously described^14^. Then, PLVs were averaged over frequencies of the desired bands.brahim

We analysed the signal from 300 seconds before seizure onset to 300 seconds after seizure onset. We determined 4 periods: inter ictal periods (at least 2 hours before seizure); pre ictal far from the beginning of a seizure (300 seconds before the beginning of the seizure, “PreIctal300s”); pre ictal, near the beginning of the seizure (10 seconds before the beginning of the seizure, “Preictal10s”), and ictal period.

For the exploration of high/low frequency bands, low frequencies were selected from the interval of frequency values from 1 to 12 Hz, while high frequencies were selected from the interval of frequency values from 30 to 70 Hz.

The target electrodes into the SoZ were those in which electrical changes were firstly identified in the beginning of the seizure. The electrodes in the NoZ (non onset epileptic zone) were those that did not engaged in epileptiform activity during the seizure and were located far from the SoZ.

We built a similarity index (SI) which measures how well the PLV in the pre ictal period allows to distinguish the involved electrodes from the rest. Thus, for each electrode we computed the PLV as described above, taking the delta band (1–4 Hz) as the low frequency band and then theta (5–8 Hz), alpha (9–12 Hz), beta (13–30 Hz), gamma (30–100 Hz) and high gamma (80–150 Hz) as high frequency bands (in total we define 5 low/high frequency combinations). We computed the average PLVs for segments from −300 s to −100 s in each electrode and each low/high frequency combination, obtaining a 5-dimension vector *V* for each electrode. Then, we performed a clustering analysis using K-means and assuming *K = 3* clusters. For each resulting cluster we obtain a centroid and the Euclidean distance *d*_*i*_ between this centroid and the centroid of the vectors for the group of involved electrodes (*V*_*c*_).

2 $${d}_{i}=\Vert {V}_{i}-{V}_{c}\Vert $$

Then, SI was computed as:

3 $$SI=1-\frac{{d}_{i}}{[{\sum }_{j\ne i}{d}_{j}]/4}$$

where *i* = argmin(*d*_*k*_) is the index of the cluster that is closest to the centroid of the involved electrodes. In this way, the SI is maximized when one of the pre-ictal PLV-based clusters matches with the group for the involved electrodes and falls far apart from the other two clusters. For visualization of the clusters we performed principal component analysis over the 5-dimensional vectors and plotted the first two principal components.

As can be seen from Eq. (2), the value of SI ranges from 0 to 1. Thus, SI = 1 implies that the involved electrodes conform exactly one of the clusters found by K-means, which in turn means that the PLV value *before* seizure onset is sorting involved electrodes from the rest.

### Statistical tests

We employed the Wilcoxon signed-rank test and rank-sum test for pairwise and non-pairwise hypothesis testing, respectively.

In order to address the possibility of spurious results given the fact that seizures are not independent, we designed a non-parametric test in which differences between mean PLV values were tested against a null distribution of 500 surrogates. Thus, to test statistical significance between the mean PLV of involved and non-involved electrodes at a given time, PLV values from the two groups were shuffled within each patient. For each surrogate we computed the average difference between the shuffled groups. Then, the difference in real data was compared against this null distribution, and a p value was obtained. The same procedure was conducted for testing differences between pre ictal300 and pre ictal 10.

## Results

**Figure 1:**
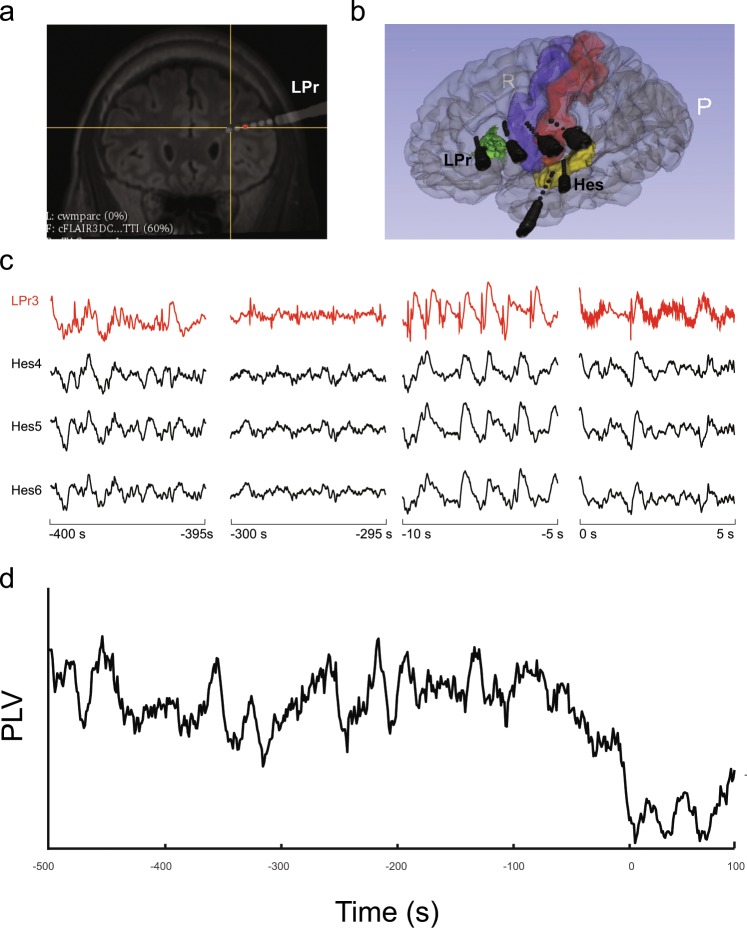
Electrode implantation and electrophysiology. (**a**) Fusion of the pre-surgery magnetic resonance (MR) and post electrode implantation cerebral tomography (CT) image (Patient 4). E1 show the position of one of the analysis electrodes (LPr electrode). (**b**) Post implantation three-dimensional brain reconstruction built from pre-implantation MR and post-implantation computed tomography (CT). The names of the electrodes are included in the image (**c**) SEEG recording of one electrode in SoZ (red) and three electrodes in NoZ. Time intervals are shown below each trace, seizure onset occurs at 0 seconds. (**d**) Phase locking value (PLV) evolution of the LPr 3 electrode within the seizure onset zone from −500 s to 100 s.

We analysed 30 SEEG recordings from 5 patients. Figure 1a shows an example of an electrode position after image processing of the pre-surgical brain magnetic resonance (MR) and the post implantation computer tomography (CT) and Fig. 1b shows a 3D reconstruction of electrode implantation for the same patient. Figure 1c shows examples of SEEG recordings of one seizure. It can be seen three pre-ictal periods and the dramatic change in frequency rhythms at ictal onset.

We focused our analysis on the coupling dynamic between the phase of low frequencies and the amplitude of high frequencies. We employed the phase locking value (PLV), which allows to fully separate phase effects from amplitude effects as a PAC measure. In Fig. 1d we show the PLV between the phase of delta band and the amplitude of gamma band for the electrode shown in red in Fig. 1c (LPr 3). As can be observed, PLV slowly decreases as the seizure onset approaches without significant visual changes in the signal SEEG signal. Indeed, as soon as the seizure starts a sudden decrease in PLV occurs.

**Figure 2:**
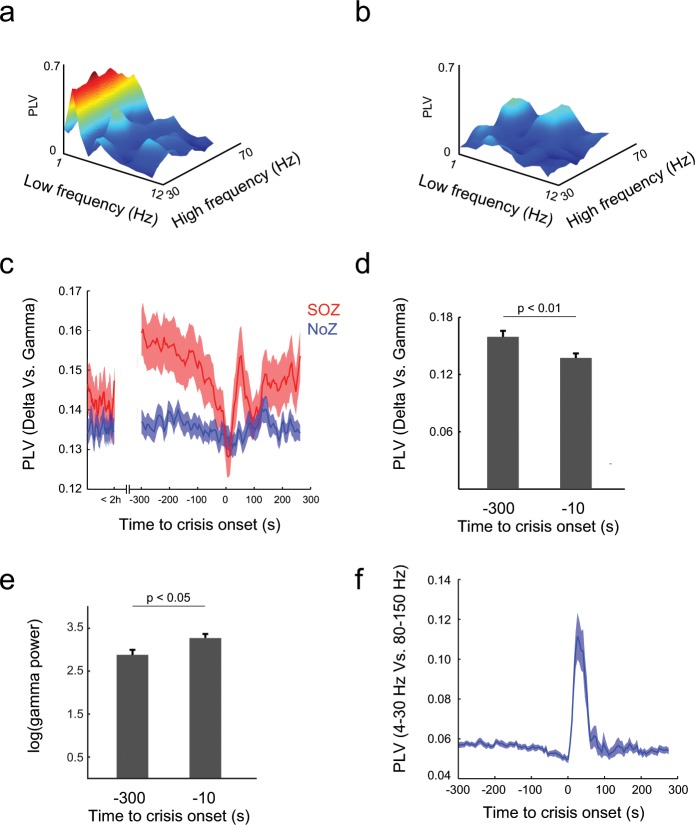
Phase locking value drop preceding seizure onset. (**a**) Phase locking value (PLV) during pre ictal far to the beginning periods (PreIctal300s). The phase of waves with frequencies around 2 Hz locks the amplitude of higher frequencies (30Hz-70Hz). (**b**) PLV decreases as the window of analysis approaches the seizure onset (PreIctal10s). (**c**) Average PLV for all epileptic seizures and patients. Locking between delta (1Hz-4Hz) and gamma (30Hz-70Hz) was low two hours before seizures onset, but it is higher for involved than for non-involved electrodes at preIctal300. A sudden decrease near the seizure onset can be seen, reaching a minimum immediately after the onset of the epileptic seizure. PLV from electrodes far from the seizure onset zone (SoZ) remains unaffected. (**d**) Phase locking becomes high between the phase of low frequencies band (4Hz-30Hz) and the amplitude of high gamma waves (80Hz-150Hz) immediately after seizure onset. (**e**) Gamma power increases as the time of seizure onset approaches (p < 0.05). (**f**) Phase locking values during ictal periods are higher than pre-ictal periods (p < 0.01, Wilcoxon signed-rank test).

We then reasoned that these sudden changes in the coupling between low and high frequency bands could indicate the arrival of a seizure onset. To test this hypothesis, we measured the PLV between low frequencies (1 Hz to 12 Hz) and high frequencies (30 Hz to 70 Hz), pooling all the seizures recorded from the five patients. Figures 2a,b show an example of the PLV in a segment (length 1 min) five minutes before the seizure onset (PreIctal300s) and the same measurement for a segment (length 1 min) immediately preceding the seizure onset (PreIctal10s), respectively. It can be seen a strong decrease in the coupling between a narrow band of low frequencies (around 2 Hz) and almost the entire band of high frequencies. To further understand the dynamics of the phenomenon we focused on the interaction between the phase of the delta band and the amplitude of the gamma band (Fig. 2c). The PLV remains low during the inter ictal period. It increases at preIctal300 to suddenly decrease when the seizure onset approaches. Thus, when compared across seizures, PLV was higher at preIctal300 than at preIctal10 (p < 10^−4^, Wilcoxon signed-rank test; p < 0.001, shuffling test, see Methods). Further analysis revealed that PLV between 4–30 Hz and 80–150 Hz suddenly increases after seizure onset (Fig. 2f), consistently with results shown in previous works^2^. To assess whether these results were no an artifact due to a decrease in the power of the gamma band, we measured gamma power and found that it was lower at preIctal300 than preIctal10 (Fig. 2e; p < 0.05, Wilcoxon signed-rank test).

We verified if the observed decrement in PLV is specifically related to the SoZ by comparing the dynamic of PLV between delta and gamma bands for the electrode in the SoZ and that in the NoZ. The PLV was higher for the SoZ electrode than for the NoZ electrode during a pre-ictal window 300 s before seizure onset (p < 10^−4^, Wilcoxon signed-rank test). However, PLV in the SoZ electrode slowly decreased as the seizure onset approached and no difference was found between SoZ and NoZ electrodes 10 s before the seizure onset (p = 0.37, Wikcoxon rank-sum test).

**Figure 3:**
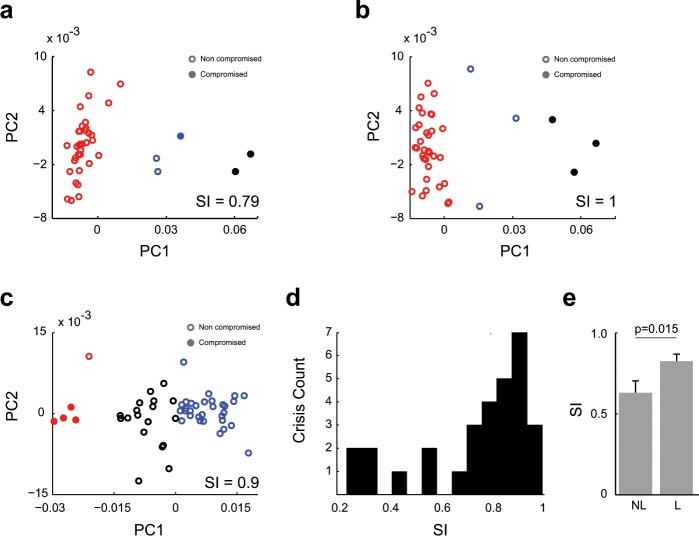
Principal component analysis of PLV vectors. (**a**–**c**) PLV between delta and the remaining bands (theta, alpha, beta and gamma). Distribution of samples on the PC1-PC2 plane (colours indicate the K-means cluster). Electrodes in the epileptic zone belong to a separated group. (**d**) Distribution of Similarity Index (SI). SI values tend to 1 as the group of involved electrodes get closer to one of the K-means clusters. (**e**) SI for patients with localized (L) and non-localized (NL) epilepsy, SI was higher in the group of localized epilepsy patients (p = 0.015, Wilcoxon signed-rank test).

We then asked whether the dynamics of frequency coupling in the entire set of electrodes was enough to discriminate the SoZ from the NoZ electrodes during the pre-ictal period (300 s to 100 s previous to seizure onset). We defined a 5-dimensional vector space in which a vector *V* was computed for each electrode, and then we applied a non-supervised clustering method to separate electrodes in the vector space in three different groups (see methods). A selectivity index (SI) was computed in order to know how far in the vector space, electrodes in the SoZ are from those in the NoZ. Figure 3a–c shows the two first principal components (PC) in three examples taken from three different patients. As can be seen, we found good separation between the electrodes located in SoZ and NoZ. Figure 3d shows the histogram of SIs for all patients and seizures. Most of the SI values were shifted to 1, indicating that in the majority of the seizures the involved electrodes tend to form a separate cluster of electrodes in the 5-dimensional vector space. Moreover, SI values were higher for those patients whose SoZ could be reliably localized and had undergone resective surgery (p = 0.015, Wilcoxon rank sum test), see Fig. 3e.

## Discussion

Mounting evidence has found brain waves synchronization as a valuable indicator of epileptic seizures. In this work we asked whether a specific kind of synchronization (phase-amplitude coupling - PAC) could aid in the characterization of seizure onset. Past efforts have focused on the study of synchronization during the seizure. For example, Weiss *et al*. showed that PAC between high gamma (80 Hz to 150 Hz) and a low frequency band (4 Hz and 30 Hz) increases late after seizure onset, and more importantly, that the topography of the phenomenon is predictive of the success of the resection surgery^2^.

In this study we chose the phase locking value (PLV) as a measure of PAC and employed this measure to study the neural dynamics before and during the seizure. Morman *et al*. have shown that a drop in synchronization may precede the high synchronization commonly observed during a seizure^12^. Yet, in Morman’s work, synchronization is assessed between pairs of electrodes and within a relative broad band (0.5 Hz to 85 Hz). In contrast, we have carried PLV measurements in each electrode separately over the whole frequency spectrum and found that PLV exhibits a dramatic drop before seizure onset, specifically for synchronization between the phase of the delta band and the amplitude of the gamma band. Notably, we observed this desynchronization in electrodes placed in SoZ that, 300 s before seizure onset, were highly synchronized. Such basal synchronization is reminiscent of the highly synchronized activity observed in interictal periods during sleep^15^. In this manner, by analysing the transition between pre-ictal and ictal activity, our work brings together previous results and suggests a scenario in which neural activity in epileptic zone is highly synchronized during the pre-ictal period and this synchronization decreases as the ictal beginning approaches.

Although our results might sound paradoxical given that in the visual analysis of the EEG signal seizures are characterized by high levels of synchronization. Computational modelling has allowed to reconcile these two seemingly opposed concepts, by showing that an increment in inhibition may lead to a desynchronization followed by seizure-like activity^16^. Desynchronization before seizure was also observed in chemoconvulsant-induced models of epileptic seizure, where the seizure emerges from a small group of highly synchronized neurons surrounded by a majority of neurons with their firing activity desynchronized^17^.

Electrode-wise analyses are desirable if the aim is to find biomarkers of the epileptic focus. In this regard we have defined a Similarity Index (SI), which quantifies how well pre-ictal PLVs between delta and a set of high frequency bands allow to individualize electrodes inside SoZ in the SEEG signal. We found that SI values were near 1, indicating a good separation of the electrodes belonging to the SoZ from those of the NoZ.

Our results are particularly interesting if we consider that recording electrodes gave valuable information about the incoming seizure long before it becomes evident in the EEG signal. In this respect, some works showed that epileptic seizures could be predicted up to 20 minutes before its occurrence^18^, proving that pathological dynamics long precedes the traditionally defined seizure onset based on expert observation of EEG recordings. Notwithstanding, these works heavily rely in machine learning techniques, which make use of several complex feature extraction methods, such as wavelet decomposition and correlation dimension estimation, resulting in powerful methods to predict seizure onsets at the expense of a reduction in biological interpretability. To which extent the abstract features obtained by automatic approaches are related to the more biologically relevant PLV measure is a matter of future work.

Altogether our results give a more complete picture of the synchronization phenomena surrounding the onset of seizures, pointing to a fundamental role of phase-amplitude coupling for understanding seizure dynamics, with emphasis in seizure prediction and epileptic focus localization. Future work will point to incorporate high frequency oscillations to improve the detection of both seizure onset zone and occurrence, and to assess how pre-ictal PLV predicts resection surgery success.
